# The Fox and the Grapes—How Physical Constraints Affect Value Based Decision Making

**DOI:** 10.1371/journal.pone.0127619

**Published:** 2015-06-10

**Authors:** Jörg Gross, Eva Woelbert, Martin Strobel

**Affiliations:** 1 Department of Cognitive Neuroscience, Maastricht University, Maastricht, The Netherlands; 2 School of Business and Economics, Maastricht University, Maastricht, The Netherlands; Max Planck Institute for Human Cognitive and Brain Sciences, GERMANY

## Abstract

One fundamental question in decision making research is how humans compute the values that guide their decisions. Recent studies showed that people assign higher value to goods that are closer to them, even when physical proximity should be irrelevant for the decision from a normative perspective. This phenomenon, however, seems reasonable from an evolutionary perspective. Most foraging decisions of animals involve the trade-off between the value that can be obtained and the associated effort of obtaining. Anticipated effort for physically obtaining a good could therefore affect the subjective value of this good. In this experiment, we test this hypothesis by letting participants state their subjective value for snack food while the effort that would be incurred when reaching for it was manipulated. Even though reaching was not required in the experiment, we find that willingness to pay was significantly lower when subjects wore heavy wristbands on their arms. Thus, when reaching was more difficult, items were perceived as less valuable. Importantly, this was only the case when items were physically in front of the participants but not when items were presented as text on a computer screen. Our results suggest automatic interactions of motor and valuation processes which are unexplored to this date and may account for irrational decisions that occur when reward is particularly easy to reach.

The Fox and the Grapes—

How Motor Constraints Affect Value Based Decision Making

*Driven by hunger*, *a fox tried to reach some grapes hanging high on the vine but was unable to*, *although he leaped with all his strength*. *As he went away*, *the fox remarked*, *“Oh*, *you aren't even ripe yet*! *I don't need any sour grapes*.*”*

*(Aesop's fable)*

## Introduction

Every day we make choices between different options based on our preferences. Research on decision making suggests that this is accomplished by assigning a subjective value to each decision option [1,2]. However, there is ample evidence that human decisions are influenced by seemingly irrelevant aspects of the choice situation [3]. One fundamental question in decision making research is therefore how humans compute the values that guide their decisions. Recent studies suggest that the subjective value of a good may depend on how this good is presented to the person at the time of the decision.

For example, Reb and Conolly [4] asked subjects how much money they would exchange for chocolate bars and coffee mugs. Subjects were either told that they owned the good, or not, and the good was either physically in front of the subject or not. The physical presence of the good, but not ownership status, significantly increased the monetary value the subjects assigned to the goods. A similar result has been observed by Knetsch and Wong [5]. The authors randomly assigned one of two goods to subjects, and asked them whether they would like to swap it for the other good. Some subjects were physically possessing the good at the time of decision, others not. Subjects who were in physical possession of the good were biased towards keeping it, whereas subjects that were not in physical possession of the good were equally likely to keep it than to swap it for the alternative. In a study by Bushong et al. [6], participants were asked how much they would be willing to pay for different snack food items under several display conditions. Subjects were willing to pay less when items were presented as pictures or words on the computer screen than when the items were physically present. This lower willingness to pay was also observed in a fourth condition when snack food items were physically present (at the same distance as before) but put behind a 9ft by 9ft Plexiglas barrier. Thus, it seems that the physical presence of a good increases its valuation, but only if the good is within immediate reach.

In any of these experiments, obtaining an item was solely based on the stated preferences. According to normative theories of decision making [7,8], the physical presence of the object is an irrelevant detail of the choice situation and should not influence the valuations or decisions. Therefore, the results of these experiments are quite puzzling. In this study we propose and test an interpretation of these findings, drawing from theories and empirical observations in the field of grounded cognition. Theories of grounded cognition posit that processes like memory, language, perception and decision making are not executed in encapsulated modular systems but are deeply interrelated [9–11]. For example, a substantial body of research suggests that the visual perception of our surrounding is not just the product of the visual information entering the visual stream but also nonvisual factors like the perceiver's physiological state, capabilities, and intentions [12]. It has been demonstrated that hills appear steeper when subjects are fatigued [13], or are wearing a heavy backpack [14, but see also 15]. Likewise, older people perceive walkable distances as longer than younger people [16], and distances appear larger when overcoming them is associated with higher effort for the perceiver [17–20]. If, on the other hand, a tool is available that facilitates reaching an object, a decrease in distance to this object is perceived [17,21].

Based on these empirical findings it has been argued that judgments of physical properties of the surrounding environment, such as length, height, and slope, are influenced by the motor actions that would be afforded to overcome them [22,23]. Climbing a hill is energetically costlier when burdened with heavy load. According to Proffitt [24], this burden is automatically integrated in how we perceive the hill. When energetic resources are scarce or effort costs are high, hills appear steeper and distances wider. Thus, our representation of the world is to some degree altered by transient changes in our capabilities to interact with it and the anticipated metabolic costs of doing so [12,23,25].

It seems plausible that not only perceptual judgments, but also preferences and decisions may be influenced by bodily states. In line with the broader literature on embodiment of attitudes and emotions [26–28], it has been shown that items are evaluated more favorably when the bodily state signals agreement (e.g. nodding the head), or approach (e.g. flexing the arm), both of which frequently co-occur with positive stimuli [29–33].

However, there is little work on how the manipulation of the physical state influences the perception of value and decision making [34]. In one of the few studies directly manipulating the physical state of objects, participants had to pick up and move one of two kitchen utensils (e.g. a fork or a pizza cutter) placed in front of them. Some objects were placed with the handle pointing towards the participant while others were placed in the opposing direction. When participants were instructed to move the object they liked more, objects where the handle pointed towards them were chosen significantly more frequently compared to a condition in which they were instructed to move the less preferred of the two objects. This suggests that stated preferences about objects are influenced by how easy or difficult it is to interact with them.

From the grounded cognition perspective outlined above, anticipated effort costs could affect the valuation process in a similar fashion as perceptual judgments. The decrease in willingness to pay Bushong et al. [6] observed when placing a barrier between the participant and the object could then be explained as follows: An object that is behind a physical barrier is more costly to reach. It takes more effort to make a grasping action circumventing the obstacle and therefore items placed behind a barrier would be perceived as less valuable, even when obtaining the item does not require any movement.

Here we test this hypothesis by directly manipulating the effort it would take to physically obtain an item. More precisely, we test whether increasing the effort it would take to reach for an item leads to lower valuations even when reaching is not required.

## Materials and Methods

### Participants and materials

In individual sessions, 54 undergraduate students were presented with 44 different snack food items consisting of candy bars (e.g. “Mars” or “Snickers”), potato chips (e.g. “Lays”), gummibears, crackers, nut mixes, or liquorice with the chance to buy one of the items at the end of the experiment. All of these items were available at local supermarkets or convenience stores. The study protocol was approved by the Ethics Committee of the Faculty of Psychology and Neuroscience at Maastricht University, and all participants gave written informed consent. Participants were recruited from the subject pool of the behavioural and experimental economics lab (BEElab) and were invited via e-mail using the software ORSEE [35]. Before starting the experimental task, participants were endowed with €14.50 to compensate them for participation in the experiment and to make sure they had money to spend on snack food.

Since we were interested in measuring the value participants assigned to different snack food items, it was important that participants liked snack food and were motivated to obtain some during the experiment. The invitation e-mail therefore clearly stated that the experiment involved snack food and that one part of the compensation for participation was monetary, and another could be in the form of snack food. To further ensure that participants were motivated to obtain snack foods, they were asked to refrain from eating for 3 hours prior to the beginning of the experiment. Participants were also instructed that they would be asked to stay in an adjacent room for 30 minutes after completion of the task. During these 30 minutes they were only allowed to eat whatever snack food they bought in the experiment. This was done to limit the influence of the market price at which participants could acquire the snack food item immediately after the experiment, and is standard practice in incentivized decision experiments [6,36]. Before the actual task, participants received detailed instructions, comprehension questions, and practice trials to ensure good understanding of the task (see S1 and S2 Figs). After reading the instructions and answering the comprehension questions and before entering the main experiment, the participants further finished five training trials with the experimenter to ensure that the Becker, DeGroot and Marschak auction was well understood. In each training trial the participant had to determine a hypothetical willingness to pay for a (non-food) good, roll the dice to determine a hypothetical price and explain whether, given the stated willingness to pay and the randomly determined price, he would buy the good and, if so, at what price.

### Procedure

Upon completion of the instruction and training part, snack food items were presented one by one to the participant. For each item, participants first indicated the maximum amount of money between €0 and €4 they would be willing to pay to receive this item after the experiment. We used a Becker, DeGroot and Marschak auction [BDM, 37] and each willingness to pay stated by the participants was potentially relevant for their outcomes (earnings and snackfood).

The BDM auction was implemented as follows: At the end of the experiment one of the 44 items was randomly selected by letting the participant draw a numbered card out of a deck of 44 cards. For the selected snack food item a selling price between €0 and €3.99 was randomly determined by the participant by rolling three dice (one four sided die and two ten-sided dice). If the stated willingness to pay was below the randomly determined selling price, the participant did not buy the good. If the stated maximum willingness to pay was equal to or above the selling price, then the participant bought the good from us at the randomly determined selling price.

With this mechanism, it is in the participants’ best interest to state their true maximum willingness to pay. This is because with their stated willingness to pay participants could only influence the chances to buy the item but not the selling price. Stating a higher amount than the true willingness to pay makes it more likely to buy the item, but in all additional cases the selling price is above the true willingness to pay. In this case the participants have to buy although they do not want to buy. In a similar manner understating the true willingness to pay may lead to situations where the item is not bought, despite the selling price being lower than the true willingness to pay.

The BDM mechanism is widely used in decision making research [36,38]. It resembles a real buying decision by measuring how much money participants are willing to give up to attain an item. Willingness to pay is our main measure of interest.

Participants also provided psychological measures of subjective value, namely liking (“Please imagine you would eat this item right now. How much do you think you would enjoy it?”), and wanting (“How much do you want to receive this item at the end of the experiment?”) ratings on a four point Likert scale. It has been suggested that liking and wanting are closely related, but neurobiologically dissociable constructs [39,40]. We were therefore interested to see whether they might be differentially affected by the weight manipulation. Note that, even if this is the case, willingness to pay, liking and wanting should all reflect the subjective value of an item, and we expect them to be highly correlated.

In addition, participants indicated the familiarity (“How well do you know this item?”) of each item on a four point Likert scale. Familiarity was measured to be able to control for the fact that subjects might exhibit a higher valuation for more familiar items. Participants saw each item only once, and always made their willingness to pay decision first, followed by the three remaining questions in random order. Subjects were encouraged to answer spontaneously, but there were no time restrictions and subjects could complete the task in their own pace.

After the evaluation of all 44 snack food items, participants provided demographic information, answered questionnaires on food craving and impulsiveness for exploratory purposes, and were asked to indicate what they thought was the purpose of the weight condition and the hypothesis of the experiment. Before leaving the laboratory, subjects were debriefed about the purpose of the experiment.

### Experimental Manipulations

#### Physical effort

**Fig 1:**
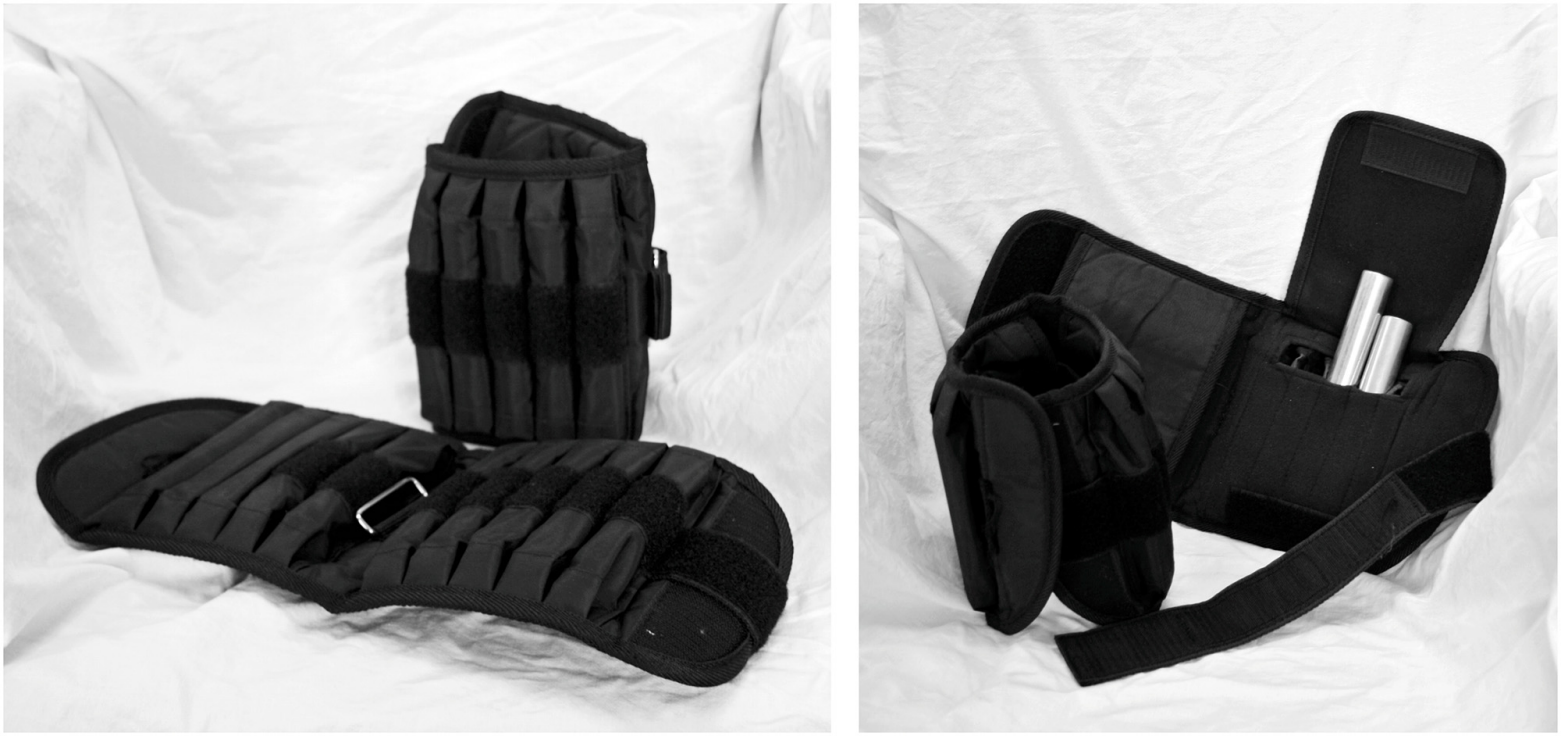
Wristbands used in the experiment. In the weight condition each wristband held ten metal bars, which add up to 4.5 kilograms (9.9 pounds) of weight. In the no weight condition all metal bars were removed.

Within subject, we manipulated the effort associated with making a reaching movement by attaching wristbands around the lower arms of our participants (see Fig 1). No explanation was given about the nature or the purpose of the wristbands. Each wristband could hold 10 metal bars, weighing 4.5 kilograms (9.9 pounds) in total (see Fig 1). For half of the items, each participant wore the wristbands with the bars (weight condition), for the other half of the items they wore the wristbands without the bars (no weight condition). Participants were instructed to sit such that the elbows were supported by the armrests of the chair and the wrists rested on their thighs. This was done to ensure that they were aware of the weight, but did not exert effort to hold the weight. The wristbands with weight would have made it significantly more demanding for the participants to grasp an item placed in front of them, but at no point during the experiment did they actually reach for an item, nor were they instructed to imagine any movements. Participants were provided with a small numeric keypad they held in their hands, which allowed them to enter their willingness to pay, liking, wanting and familiarity ratings without moving their arms.

The order of the weight conditions was counterbalanced across participants. To create two comparable sets and reduce the influence of strong preferences for certain types of snack food (like chocolate, liquorice or fruit gums), the allocation of the 44 snack food items to the weight and no weight condition was not fully randomized. Instead, we formed pairs of similar snack foods (like two types of chips or two different chocolate bars) and never presented both elements of a pair in the same weight condition (see S3 Fig and S1 Table). This ensured that the same amount of each category of snack foods was presented in both conditions to the participant, and mitigated the effect of potentially strong preferences for specific categories of snack food on the mean willingness to pay per condition. Which particular chocolate bar or chips bag was presented in the weight or no weight condition was randomized across participants.

#### Reachability

Between subjects we manipulated the reachability of the items. Participants in the physical condition had each food item placed physically in front of them at the time of decision making. Thus, during value judgements, the item was reachable with a simple arm movement. In the control condition, which we call the computer condition, items were not physically present and thus not physically reachable, but were presented to the participants as text on the computer screen. Participants in the computer condition were instructed that snack food items were normal package sizes, as available in the supermarket. The same items were used in the computer condition and the physical condition, with the exception of five items that were not available in stores when the computer condition was run. These were replaced with similar items. Note that our primary interest lies in the within-subject comparison.

When the snack food is physically reachable, it is possible to physically obtain the item with an arm movement. This arm movement would be more effortful when wearing the heavy wristbands. If these anticipated effort costs influence the valuation process, subjective valuation and willingness to pay in the physical condition will be lower in the weight condition compared to the no weight condition. In the computer condition items are not reachable by arm movements and thus not physically obtainable. Therefore, the increased effort of making an arm movement by having heavy wristbands around the lower arm should not influence the valuation process, and we expect no difference of our physical effort manipulation on valuation in the computer condition.

While it might seem a more natural choice for the control condition to present pictures of the snack food items on the computer screen, we chose not to do so because pictures of physical stimuli have some spatial properties, and have been found to affect cognitive processes based on the depicted physical properties [41], whereas this seems not to be the case for words [42]. Also note that any confounding effect of weight on value judgments, such as stress or discomfort should still be present in the computer condition, while an effect that is specific to anticipated effort of reaching for the snack food should not.

### Data analysis

From each subject, we obtained responses for 44 snack food items, 22 in the weight condition and 22 in the no weight condition. Thus, our data set is hierarchically structured: Responses for individual items are nested within subjects. Unlike what is frequently done in the experimental psychology literature [43], we chose to refrain from aggregating the data on the subject level and instead report the results of a multilevel regression model which we describe in detail below. By refraining from aggregating the data on the subject level, we are able to include control variables on the item level (in our case the familiarity of the item). In the regression model we also control for possible order effects of the weight manipulation.

The regression model needs to account for the fact that data points belonging to the same subject are not independent. Therefore we fitted a random intercept regression model of the following form:

$$y_{ij}=\;u_{j}+\;\beta X_{i}+\;e_{i},\;\text{where }\;u_{j}\;\sim \;N\left(\mu_{u},\;\sigma_{u}^{2}\right)$$ (1)

where *y*_*ij*_ is observation *i*, belonging to subject *j*, *u*_*j*_ is the estimated individual-specific intercept of subject *j*, *X*_*i*_ is the vector of predictors, and *e*_*i*_ is the error term belonging to observation *i*. The subject specific intercept accounts for the possibility that some subjects show systematically higher or lower responses than others [44,45]. Additionally, we account for possible further dependencies within subjects, as well as for possible heteroscedasticity, by using cluster-robust standard errors [46].

We specify the model with the following predictors:

$$\begin{array}{l} y_{ij}=\;u_{j}+\;\beta_{1}\;\times \;physical_{i}+\;\beta_{2}\times \;weight_{i}+\;\beta_{3}\;\times \;physical_{i}\;\times \;weight_{i}\;\\ +\;\beta_{4}\;\times \;familiarity_{i}+\;\beta_{5}\;\times \;order_{i}+\;\beta_{6}\;\times \;order_{i}\;\times \;weight_{i}\\ +\;\beta_{7}\;\times \;order_{i}\times physical_{i}+\beta_{8}\;\times order_{i}\;\times \;physical_{i}\;\times \;weight_{i}\\ +e_{i},\;\;\;\;\text{where }u_{j}\;\sim \;N\left(\mu_{u},\;\sigma_{u}^{2}\right) \end{array}$$ (2)

*Weight* is a dummy variable indicating whether an observation belongs to the weight or the no weight condition (0 = no weight, 1 = weight), *physical* is a dummy variable indicating whether an observation belongs to the physical or the computer condition (0 = computer condition, 1 = physical condition). As a result of the way the dummy variables are coded, the intercept of the model (*μ*_*u*_) corresponds to the combination: no weight, computer condition. *β*_*2*_ indicates the effect of weight in the computer condition only, and *β*_*3*_ indicates by how much the effect of the weight differs between the computer and the physical condition. The effect of the weight in the physical condition is therefore given by *β*_*2+*_ *β*_*3.*_ We test for the significance for this combination of coefficients using a Wald test.

We include a set of control variables: *familiarity* is a continuous variable indicating the familiarity of the item as reported by the subject and *order* refers to the order of the weight conditions (0 = starting without weight, 1 = starting with weight). To control for the fact that the effect of weight might differ depending on whether a subject started with the weight condition or the no weight condition, we include the interaction term of *order × weight*, as well as interaction terms up to the three-way interaction of *order × physical × weight*.

As a measure of effect size we report d, computed by dividing the regression coefficient by the item-level standard deviation obtained in the respective regression model [47]. We fitted the regression model using the xtreg command in Stata 10 [48], additional analyses were carried out using R [49]. Separate regressions were performed for willingness to pay, wanting and liking ratings, each with the same set of predictors. In Supporting Information S1 we further elaborate on the analysis, and for comparison we also report the results of aggregated data t-tests and an ANOVA model on subject-level averages.

## Results

Our final data set contained decisions from 24 participants (12 female, 12 male, mean age 22.6 ± 3.0 years) in the physical condition and from 26 participants (13 female, 13 male, mean age 22.4 ± 2.7 years) in the computer condition. Two participants in the physical condition had to be excluded from the analysis because of a computer malfunction. From the computer condition two participants were excluded, one who bid zero for all items, indicating that he was not motivated to buy any snack food, the other participant reported that he changed his bidding strategy halfway through the experiment. Note that including these participants in the data analysis does not change any significance level of our hypothesis tests reported below.

### Descriptive statistics

**Fig 2:**
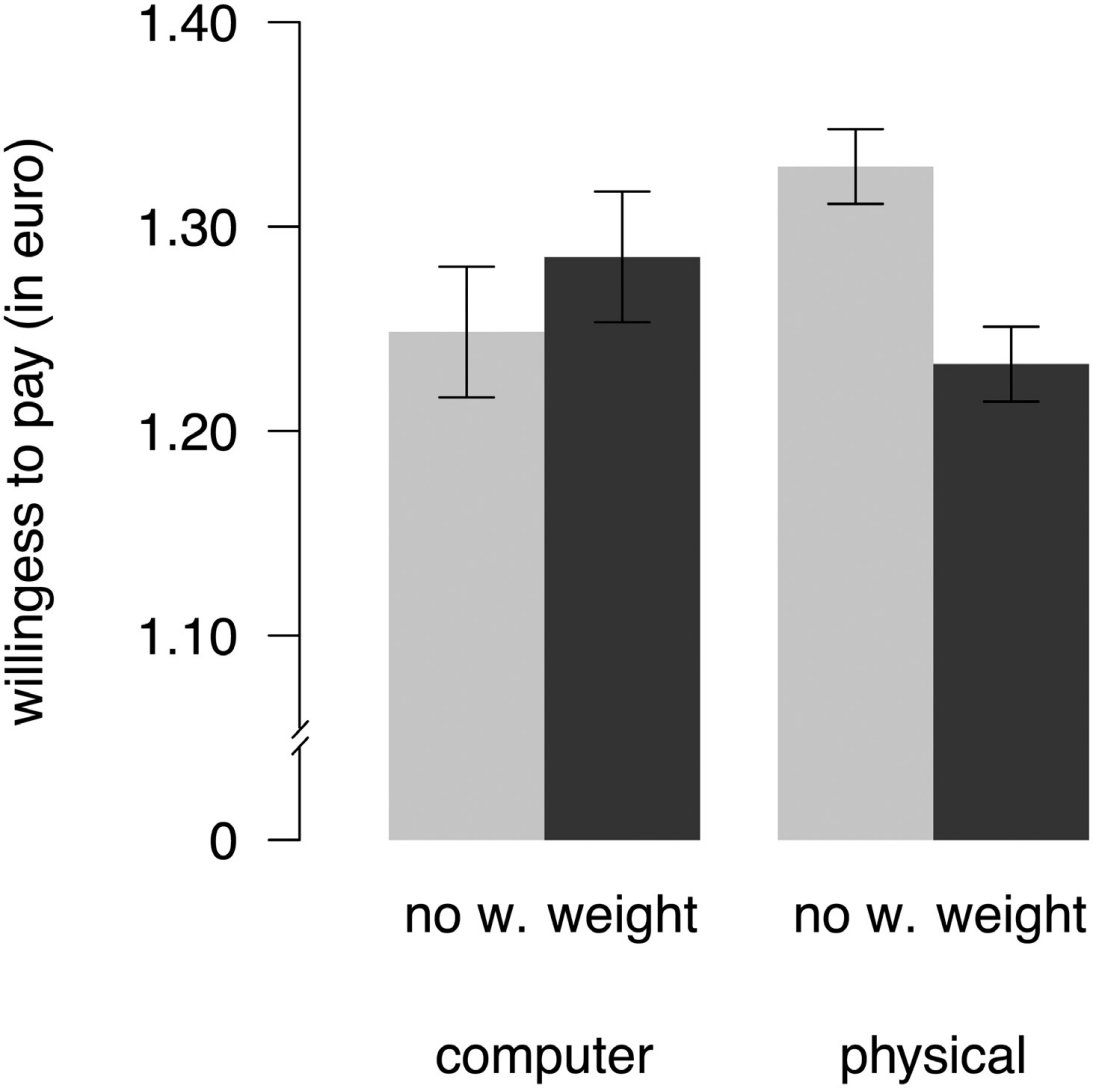
Effect of weight on willingness to pay separately for physical and computer condition. Error bars show the within-subject standard errors of the mean [50,51] and are therefore only informative for evaluating within-subject differences between wearing wristbands with weight (black) vs. no weight (grey).

**Fig 3:**
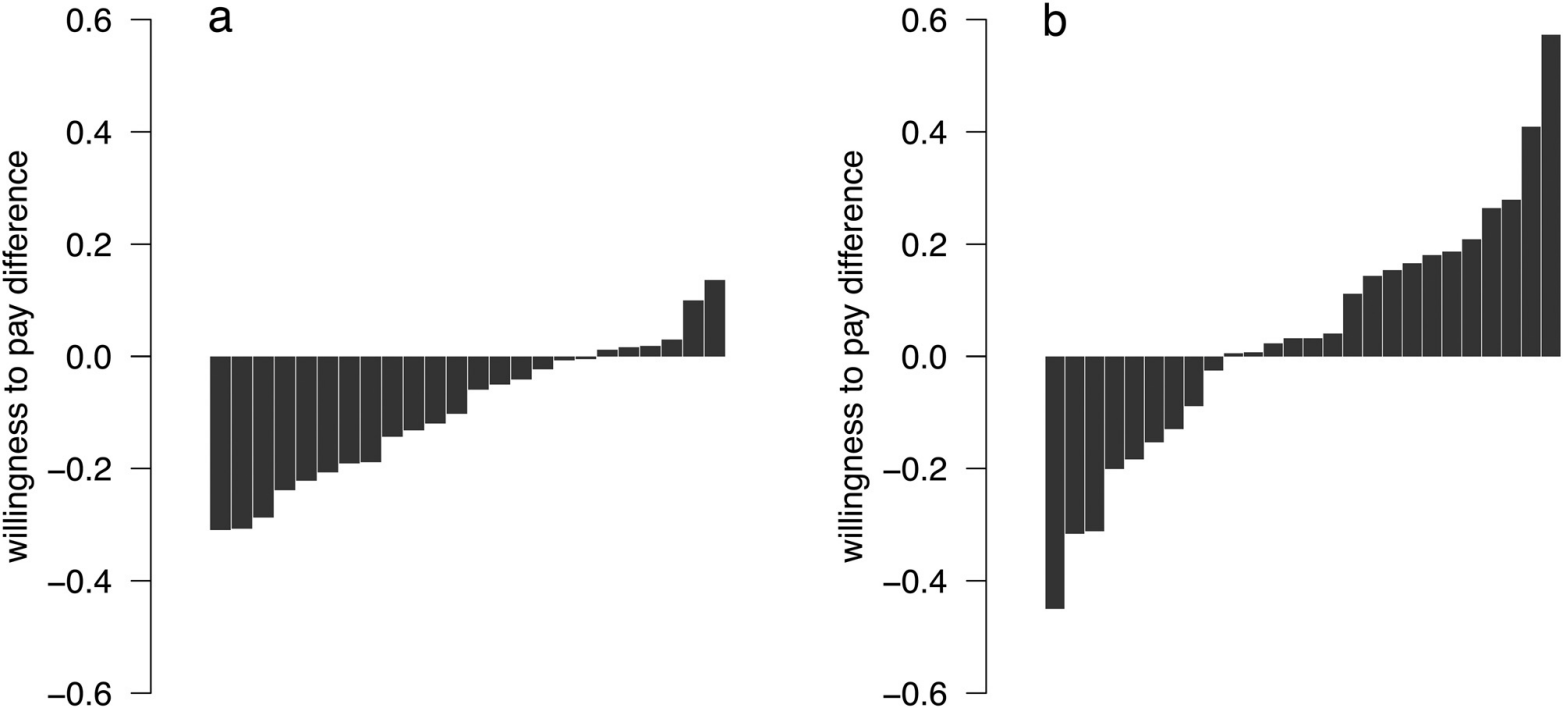
Average willingness to pay difference per individual across physical condition (a) and computer condition (b). Each bar shows the average difference in willingness to pay of one participant across weight conditions. Negative values indicate that participants were willing to pay less for items in the weight condition.

In the physical condition, when snack food items were physically in front of subjects, participants reported an average willingness to pay of €1.33 in the no weight condition. However, the same participants decreased their average willingness to pay by 10 cents when they had heavy weight on their arms (mean Cohen’s *d* = 0.26, see Fig 2). We did not observe this difference in valuation between the weight and the no weight condition when items were presented as text on a computer screen instead. Here, participants slightly increased their willingness to pay by 4 cents on average when wearing heavy weight. Fig 3 shows the difference in willingness to pay between the weight and the no weight condition for each individual subject. In the physical condition, for most subjects the willingness to pay was lower with weight than without weight, whereas this was not the case in the computer condition.

**Fig 4:**
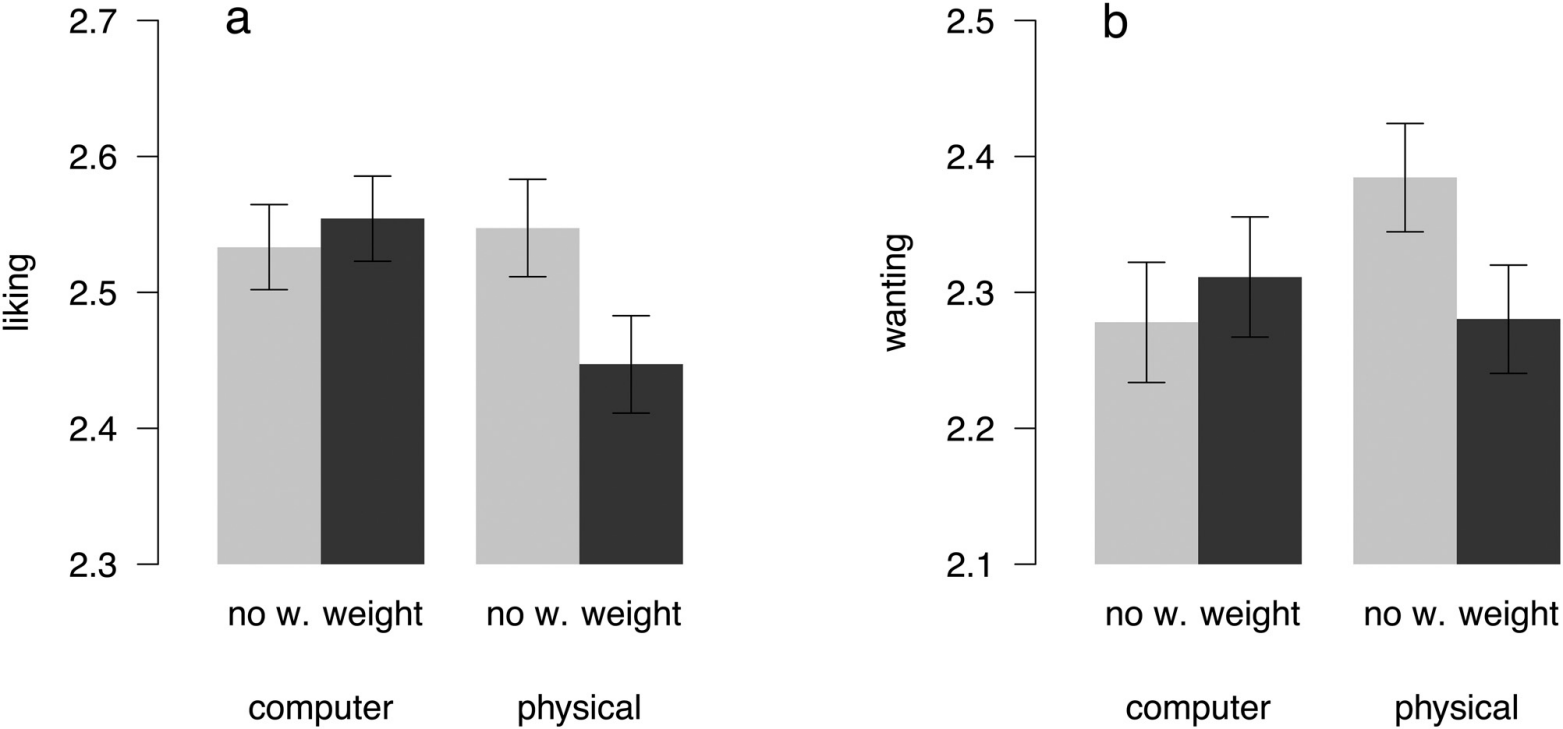
Effect of weight on liking (a) and wanting ratings (b). Error bars show the within-subject standard errors of the mean [50,51] and are therefore only informative for evaluating within-subject differences between wearing wristbands with weight (black) vs. without weight (grey).

Wanting and liking ratings were similarly affected by the weight manipulation. Participants in the physical condition reported an average liking of 1.55 without weight, but only an average liking of 1.45 with weight. In contrast, participants in the computer condition reported an average liking of 1.53 in the no weight condition and 1.55 in the weight condition (see Fig 4a). Similarly, for wanting, participants in the physical condition reported an average of 1.38 in the no weight condition, and 1.28 in the weight condition. Whereas, participants in the computer condition reported an average wanting of 1.27 without weight, and 1.31 with weight (see Fig 4b). Thus, it appears that weight decreased liking and wanting in the physical condition, but not in the computer condition. Subject-level differences in liking and wanting across the two conditions are displayed in S4 and S5 Figs.

### Regression results

To test our hypothesis, we report the results of the three separate regressions, one for each of the dependent variables willingness to pay, liking, and wanting. The details of the regression model are described above in section "Data Analysis".

For willingness to pay, we observe a significant interaction between our anticipated effort manipulation and reachability (Physical × Weight coefficient = -0.15, *p* = .02, two-sided; *d* = 0.23, see Table 1). This interaction indicates that the effect of weight on willingness to pay differs between the physical condition and the computer condition. In line with the descriptive results reported above, the regression model reveals a significant decrease of willingness to pay in the physical condition when wearing heavy wristbands (Weight coefficient + Physical × Weight coefficient, Wald test, chi2(1) = 22.21 *p* < .01; *d* = 0.20), but no significant change in valuation in the computer condition across the anticipated effort manipulation (Weight coefficient = 0.02, *p* = .76, two-sided, see Table 1). Thus, when items were physically in front of participants, wearing heavy wristbands around their arms significantly decreased willingness to pay, while this was not the case when the items were presented on a computer screen.

**Table 1:** Results of Random Intercept Regression. Dependent Variable: Willingness to Pay.

	Coef.	95% CI	p
Constant (Computer Condition, No Weight)	1.16	[0.78,1.54]	<.01
Physical Condition	0.00	[-0.50,0.50]	.99
Weight	0.02	[-0.10,0.13]	.76
Physical × Weight	-0.15	[-0.28, -0.03]	.02
Familiarity	0.13	[0.09,0.18]	<.01
Order of Weight Conditions	-0.35	[-0.79,0.10]	.12
Order × Weight	0.02	[-0.15,0.19]	.81
Order × Physical	0.20	[-0.39,0.80]	.50
Order × Physical × Weight	0.07	[-0.13,0.26]	.50
*σ*_*u*_ (SD between subjects)	0.52		
*σ*_*e*_ (SD within subjects)	0.65		

2200 trials, nested within 50 subjects. Standard errors are corrected for potential heteroscedasticity and autocorrelations at the subject level. All *p* values are two-sided. In the table, dummy variables are generally referred to with the condition coded as 1. *Order* indicates whether participant started with the weight (Order = 1) or no weight condition (Order = 0).

We obtained similar effects for the regressions on liking and wanting ratings (see S2 and S3 Tables for the full regression results on liking and wanting). The interaction of effort and reachability condition was statistically significant for wanting (random intercept regression, Physical × Weight coefficient = -0.21, *p* = .03, two-sided; *d* = 0.24) and marginally significant for liking ratings (Physical × Weight coefficient = -0.13, *p* = .08, two-sided; *d* = 0.15). However, Wald tests directly comparing weight vs. no weight in the physical condition did not reach significance for liking and wanting ratings.

### Additional analysis

To test whether willingness to pay, liking, and wanting measure closely related constructs, we computed how strongly they were correlated for each subject. Correlations were generally high (willingness to pay and wanting: mean *r* = .60, range: .-12 to .96; willingness to pay and liking: mean *r* = .57, range: -.03 to .97), and highest for wanting and liking (mean *r* = .81, range: .21 to .97). For each of the correlation types a t-test over the distribution of subject specific correlation coefficients shows that they are significantly different from zero (n = 50, p < 0.01 for each test)

**Fig 5:**
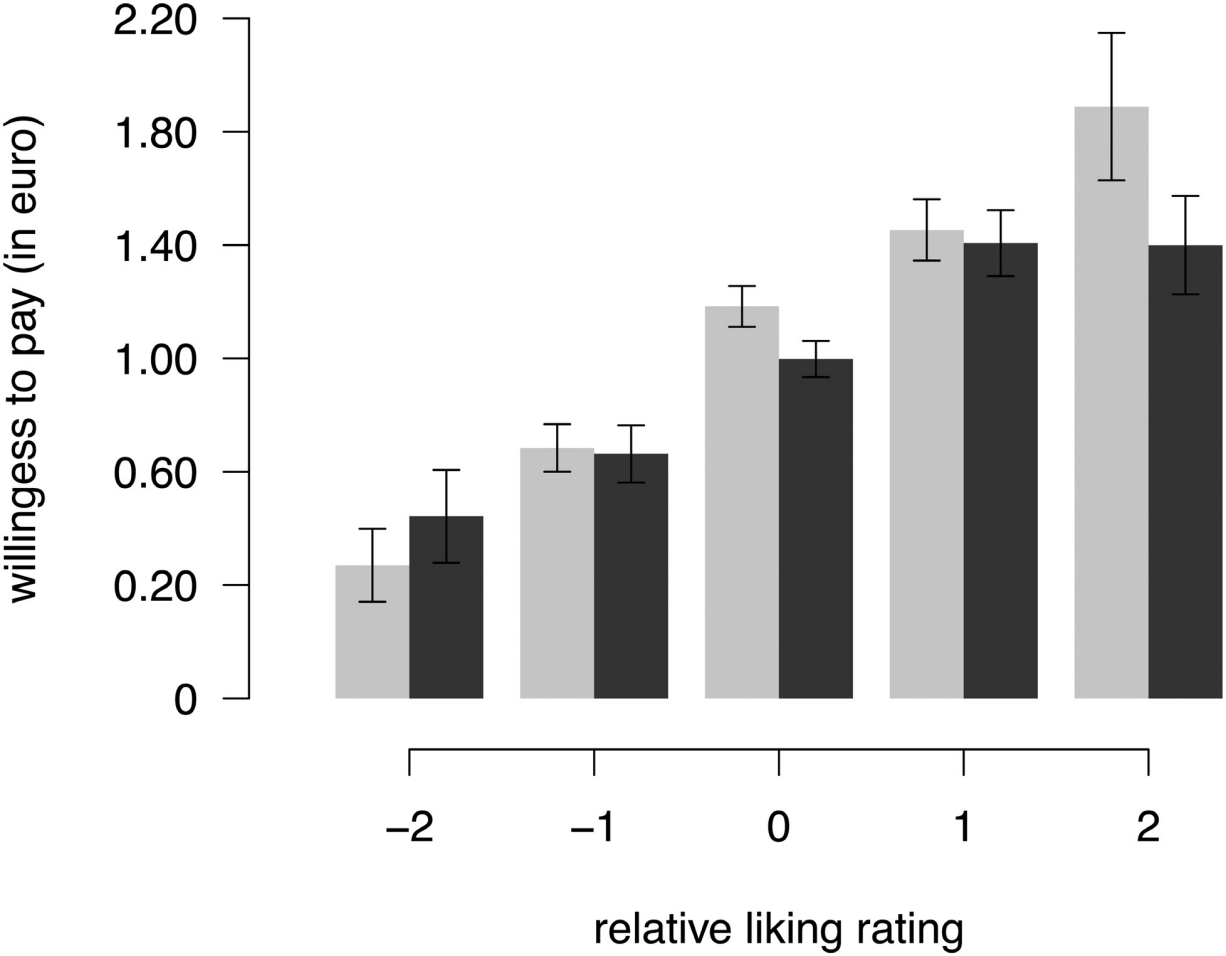
Effect of weight on willingness to pay across relative liking ratings. Liking ratings are shown as the deviation from the median rating within each weight condition. Error bars show the within-subject standard errors of the mean [50,51] and are therefore only informative for evaluating within-subject differences between wearing wristbands with weight (black) vs. without weight (grey).

Lastly, we explored whether the effect of the weight on willingness to pay in the physical condition depended on how much a participant liked an item. Since liking ratings were not measured independently of the physical effort manipulation, we sought to remove the influence of weight on liking ratings before performing the actual analysis. To this end, for each participant, we subtracted the median liking rating under each of the weight conditions from each liking rating of the participant. This way we obtained a median-centered liking rating for each item, which indicated how much this item was liked by the participant, relative the median item of the respective physical effort condition. We then compared willingness to pay between weight and no weight across the different values of these relative liking ratings. As can be seen in Fig 5, willingness to pay increases linearly with liking ratings in the no weight condition, while in the weight condition the increase of willingness to pay diminishes for highly liked items. Thus, the effect of weight on willingness to pay was more pronounced the more the participant indicated to like the item.

After the experiment, participants were asked to indicate what they thought was the purpose and the hypothesis of the experiment. In the physical condition, six subjects indicated a directed hypothesis, four of which hypothesized an effect in the opposite direction of our hypothesis. In the computer condition, four subjects indicated a directed hypothesis, two in each direction. Thus, we consider a demand effect resulting from correctly guessing the hypothesis unlikely.

## Discussion

We showed that physical constraints influence subjective value and actual purchasing decisions. Attaching heavy wristbands to participants’ arms decreased willingness to pay and subjective valuation for different snack food items, even though participants in the experiment never actually had to reach out for an item, nor were they asked to imagine any arm movements. Our findings suggest that the anticipated metabolic costs of obtaining an item are automatically integrated in the value computation process when the item is physically reachable. Liking and wanting ratings were similarly affected by the weight manipulation, and we did not find evidence that liking and wanting are dissociable constructs in this task.

Importantly, the wristbands affected willingness to pay only when goods were physically in front of the participant, and not when goods were merely presented as texts on a computer screen. Thus, our data suggest that the change in metabolic costs or effort due to the weight for making an arm movement only influence valuation when items are in fact reachable. In addition, we observed that the effect of weight on monetary valuation was most pronounced for items that were more liked.

From a normative perspective on decision making this finding is puzzling. Grasping the object was not required at any point in the experiment, and participants knew that obtaining an item was not dependent on physical effort, but only on their stated willingness to pay. Consequently, such irrelevant aspects as having weight attached to the lower arms, should not influence the decision. In the following, we seek to explain why it might be biologically plausible that the physical constraints do influence value perception nevertheless.

In this experimental setting, as is often the case in modern daily life, choices occurred dissociated from physical action. In a supermarket, whether we can afford a product does not depend on physical constraints but on the amount of money we carry in our pocket. Similarly, we can order food online, making the decision even less dependent on physical action. However, apart from modern human society, whether something is easy to obtain or not is highly relevant. For example, any foraging decision in animals involves a trade-off between the value that can be obtained and the associated metabolic costs [52]. Imagine a bear looking at a beehive high above in a tree or a group of lions tracking the movement of a gazelle. Whether it is optimal to climb the tree or to hunt the gazelle does not solely depend on the value of the goal, but also on the costs incurred by exerting the effort. When two actions yield similarly valued outcomes, the action that is less costly is the natural choice [53].

In natural environments, where anticipated effort is central to any decision processes for the computation of goal value and effort costs could be closely intertwined. As a result, computing the value of a good may be automatically influenced by the anticipated effort of obtaining the good. Something that is easy to reach would then be attributed a higher value even in choice situations in which grasping the object is not necessary to obtain it, like in a purchasing decision.

Some direct support for this hypothesis comes from an experiment by Beilock and Holt [54]. In this experiment, both skilled and novice typists made a series of binary choices between different printed letter pairs, each time choosing the letter pair they preferred. They found that only skilled typists showed a preference for letter pairs that can be easily typed, such as 'FJ', over pairs that are more difficult to type (e.g. 'FV'), whereas novices had random preferences. This effect of physical effort on preference vanished when skilled typists were instructed to hold a typing pattern in memory, presumably diminishing the capacity to compute the effort associated with typing the letter pair. Interestingly, this was only the case if the typing pattern that subjects needed to remember actually engaged the same fingers as required to type the letter pairs. This suggests that effects of physical effort on valuation and preference are indeed mediated by simulation of the relevant action.

Also, a substantial body of neurophysiological and neuroimaging data points at a strong interaction of value computation and motor-related processes [55]. The brain constantly keeps track of whether objects are within reach [56], and simulates movements that are feasible to manipulate the immediate environment, even in the absence of any intention or explicit goals [22,57–59]. Throughout the decision making process, decision-variables such as subjective value are represented not only in brain regions that have been directly linked to value computation and decision making, such as the ventromedial prefrontal cortex [36,60–62], but are also found in brain areas that are linked to motor preparation and execution [63–67]. A recent neuroimaging study lends support to our interpretation that effort is automatically integrated with value [68]. When subjects received a positive outcome after exerting either high or low effort, activity in the ventral striatum, commonly interpreted as an evaluation of the outcome in the form of a reward prediction error, was higher after low effort. Thus, effort exerted to obtain an outcome was immediately integrated with the outcome value into a net-value. Our results suggest that such integration occurs to some extent also when assessing the value of the reward before obtaining it and even when physical actions are actually not required.

It seems plausible that anticipated effort of obtaining a good is most relevant if this good is highly liked, since it is more likely that the person will actually want to engage in a physical action to obtain the good. If, on the other hand, a good is not liked, anticipated effort should be less relevant and automatic motor processes might be triggered to a lesser extent. In line with this, we found that the more liked items were more strongly affected by the weight manipulation. However, since liking evaluations were obtained during our weight manipulation, this result should be interpreted with caution. Future research should aim to confirm this finding, ideally measuring liking independently of the experimental manipulation.

Further, we did not measure neurobiological motor signals directly. An interesting next step would therefore be to assess the state of the motor system during the different conditions in our task. In humans, transcranial magnetic stimulation (TMS) offers the possibility to study activation of the motor system with good spatial and temporal specificity during decision making. Applying a TMS pulse over specific parts of the primary motor cortex leads to a measurable change in activity in the associated effector muscles. The amplitude of this so-called motor evoked potential (MEP) reflects the excitability of the stimulated part of the motor cortex at this point in time. This excitability has been found to be higher for graspable objects that are within reach [69]. Thus, we would expect higher MEPs in the physical condition than in the computer condition. Moreover, since motor involvement in the computer condition should be minimal, we would not expect any differences with and without weight. Regarding the difference between the weight and no weight in the physical condition, two different outcomes appear plausible. On the one hand, motor excitability has been found to correspond to the amount of force required for an observed action [70]. This suggests that the heavy wristband, which would make a reaching movement more effortful, should lead to higher MEPs. On the other hand, using the same TMS/MEP paradigm in the context of value based decision making, Gupta and Aron [71] have found that MEPs increase also with the offered reward size. That is, the more an individual wants to receive a certain food item, the higher the cortical excitability for arm muscles. The authors suggest that a high urge for obtaining an item results in higher motivation for movement. This finding is in line with our observation that the effect of the weight is most pronounced in highly liked items because the weight should only matter if there is a certain motivation for movement. However, given that our anticipated effort manipulation seems to decrease the desirability of the food items, this finding also suggests the weight might decrease motor excitability. In fact, it seems plausible that both of these effects take place somewhat in parallel, but—since effort computation must somehow precede the effort-induced devaluation—the good temporal resolution of the TMS/MEP paradigm (see 64) might even allow temporally dissociating them.

In summary, we show that irrelevant motor constraints affect value based decisions. Items that were more difficult to grasp were perceived as less valuable. This suggests that processes underlying the computation of value and effort are closely intertwined to an extent that anticipated effort for physically obtaining a good is automatically integrated in the value computation process, explaining why goods appear more valuable to subjects when physically present [4,5], but not if reaching for them is obstructed by a barrier [6]. This mechanism is unexplored to this date and could help to explain maladaptive human behavior that occurs when reward is easy to reach, such as overeating or impulsive purchases.

## Supporting Information

S1 Dataset Full dataset underlying the analysis. (CSV)

S1 Fig Instructions given to the participants in the physical condition. (PDF)

S2 Fig Page 2 of the instruction given to the participants in the computer condition. The other pages of the instructions were identical to the physical condition.
(PDF)

S3 Fig Stimuli used in the physical condition. Pictures of the snack food items used in the physical condition. Snack foods near to each other comprise pairs of similar items (e.g. two chocolate bars or two licorice snacks). Elements of a pair were never presented in the same condition within one participant. One was presented in the weight, the other one in the no weight condition. This way, it was assured that there were the same kind of snack foods present in both conditions. Which item was presented in which condition was randomized across participants.
(TIFF)

S4 Fig Average liking difference per individual in the physical condition (a) and computer condition (b). Each bar shows the average difference in liking of one participant across weight conditions. Negative values indicate that participants gave a lower average liking rating for items in the weight condition.
(PDF)

S5 Fig Average wanting difference per individual in the physical condition (a) and computer condition (b). Each bar shows the average difference in wanting of one participant across weight conditions. Negative values indicate that participants gave a lower average wanting rating for items in the weight condition.
(PDF)

S6 Fig Willingness to pay differences across weight and no weight condition for each item pair and individual. Each barplot shows the willingness to pay differences of one subject in the physical condition. Each bar represents the difference in willingness to pay for one item pair. As part of our item randomization strategy, we formed pairs of similar items. Within one subject, items from the same pair were never presented in the same condition. Thus, it is possible to compute for each subject the difference in WTP across the two items of each pair. It should be noted that, although items of the same pair were of the same type of snack food, they were still different, and any strong individual preferences (e.g. for or against cheese flavor on crisps, for or against orange flavor on chocolate, for or against raisins) still play a role.
(PDF)

S7 Fig Willingness to pay differences across weight and no weight condition for each item pair and individual. Each barplot shows the willingness to pay differences of one subject in the computer condition. Each bar represents the difference in willingness to pay for one item pair. As part of our item randomization strategy, we formed pairs of similar items. Within one subject, items from the same pair were never presented in the same condition. Thus, it is possible to compute for each subject the difference in WTP across the two items of each pair. It should be noted that, although items of the same pair were of the same type of snack food, they were still different, and any strong individual preferences (e.g. for or against cheese flavor on crisps, for or against orange flavor on chocolate, for or against raisins) still play a role.
(PDF)

S1 File Contains supporting information on the hypothesis test, reports the results on ANOVA models, as well as regression diagnostics. (PDF)

S1 Table Stimuli used in the computer condition. Names and descriptions (in parentheses) were presented together on the computer screen. Snack foods in the same row comprise pairs.
(PDF)

S2 Table Random intercept regression model with control variables. Dependent variable: wanting ratings.
(PDF)

S3 Table Random intercept regression model with control variables. Dependent variable: liking ratings.
(PDF)
