# Supplementary material for: The Fox and the Grapes—How Physical Constraints Affect Value Based Decision Making
Source: PLoS One. 2015 Jun 10;10(6):e0127619. doi: 10.1371/journal.pone.0127619 (PMC4464737; doi:10.1371/journal.pone.0127619)
Supplement: S1 Fig — (PDF) [file pone.0127619.s002.pdf]

Welcome,

You are participating in an experiment on decision making.
The study will last about 90 minutes.

The experiment is structured as follows:

1. You read the instructions for the experiment.
2. You answer some comprehension questions.
3. You can practice the task (5 min).
4. You do the task (35 min).
5. Your earnings are determined
6. You answer a short questionnaire (10 min).
7. You will stay in an adjacent room for further 30 min.

Please proceed to reading the instructions on the next pages.
Whenever you have a question, please ask the experimenter for clarification.

Instructions

Task
In this experiment, you will have an opportunity to buy a snack food from our store using €14.50 that you receive from us. You will receive the €14.50 once you have read the instructions and answered the comprehension questions correctly.

At the end of the experiment, you will be asked to stay in an adjacent room for 30 minutes. During this time, the only food that you will be allowed to eat is whatever snack you bought from us during the experiment.

The snack food items that are available in this experiment are real and recently purchased for the purpose of this experiment. You will see all of them during the experiment.

Your task in this experiment is to decide and tell us the maximum amount that you would currently be willing to pay for each of these items.

Round structure
The experiment consists of many rounds, all of which have a similar structure. In each round:

1. First, you will see which item is on offer in this round
2. Then you will enter the maximum amount that you are willing to pay for this item (in €). You will need to enter a number between €0 and €4. You can also enter decimal numbers like €3.47 using the dot as a decimal point.
3. You will answer a few questions about the item.

There is no strict time limit for giving an answer. Nevertheless, try to answer spontaneously, without thinking too much. After 22 of these rounds there will be a break, then there will be another 22 rounds. You will see each item only once. The items will be right in front of you so you can see them well. Please do not touch the items.

During this task a wristband will be attached to each of your arms.

Your earnings in this experiment
At the beginning of the experiment, you receive €14.50. You can use this money to buy one item of snack food from us. Whatever you do not spend remains yours, just like in everyday transactions. Although you will place bids on 44 items, you will only be allowed to buy one of them.

How do we determine whether you bought or not?
At the end of the experiment, **one** of the rounds will be randomly chosen. You will be asked to draw a card from a stack of cards numbered from 1 to 44. The round with the number that you draw will be the one that counts. Note that every round has the chance to be selected, and only one round will be selected. Therefore, you don't need to worry about spreading your €14.50 budget. In fact, you can treat every round as if it were the only round. Each time you have to bid on an item, it is in your best interest to report exactly your maximum willingness to pay for being allowed to eat this item at the end of the experiment.

After you picked a card to randomly select the round that counts, you can see which item was presented in this round and what was your bid on this item. Then, the actual price for the item will be determined randomly. Therefore, you will be asked to throw 3 dice that will determine the price. The price shown is the actual price for which you can buy the item from us. Please note that your bid does not influence the actual price of the item.

Whether you indeed buy the item from us depends on your bid and the actual, randomly determined price. If your bid is higher than or equal to the actual price (so you would be willing to pay the actual price) you will buy the item at the actual price and keep the rest of the €14.50.
On the other hand, if your bid is lower than the actual price(so you would not be willing to pay this actual price), then you do not buy the item and keep the €14.50.

Note that if you buy you never pay more than the actual, randomly determined price! If your bid is higher than the actual price you do not have to pay your bid, but just the actual price! Therefore, the best you can do in each round is simply to estimate what the item is worth to you (the maximum you would be willing to pay for it) and bid exactly this amount.

You should not bid more money on an item than you actually are willing to pay. Stating higher bids increases the chance that you will buy the item. However, the downside of this is that this involves the risk of buying the item at a price that is higher than what you are willing to pay for it.

For example: Suppose that the most you would like to pay for a bag of biscuits is €3, but in order to increase the chances of getting the biscuits you decide to bid €4. The actual price is randomly determined at €3.60. Then, you have to purchase the biscuits for €3.60, a price that is higher than what the biscuits are actually worth to you (€3).

You might think that your best strategy is to bid lower than your actual valuation for the item. This is incorrect. The price that you pay is determined by the numbers you throw with the dies and not by your bid. Bidding lower than your true value you would not affect the price that you pay, but you run the risk of not buying although the price is acceptable to you.

For example: Suppose that the maximum you would like to pay for a chocolate bar is €3.50, but in order to keep more money you decide to bid only €1. The actual price turns out to be €2. You will not buy the chocolate bar because you bid only €1. Had your bid been your true value of €3.50, you would have purchased the chocolate bar for €2 and kept €12.50 in cash.

To sum up, the best you can do in your own interest is to bid exactly the amount which you are maximally willing to pay for the item at stake.

Here are a few examples, to make this mechanism clear:

Example 1: In the round selected for payment, Manuel was bidding on chips. Manuel's bid in this round was €2.65. The randomly selected actual price turns out to be €2.00.
Manuel buys the chips because the actual price (€2.00) is lower than his maximum willingness to pay (€2.65). Manuel gets the chips and pays €2.00. He keeps €12.50 from his initial €14.50.

Example 2: In the selected round, Manuel was bidding on popcorn. Manuel's bid was €1.35. The randomly selected actual price turns out to be €4.00.

Manuel doesn't buy the popcorn because the store's price (€4) is higher than Manuel's maximum willingness to pay (€1.35). He keeps the €14.50.

Example 3: In the selected round, Manuel bid €2.80 on gummibears. The actual price for the gummibears turns out to be €2.50.
Manuel buys the gummibears for €2.50 because his bid is greater than the actual price. He keeps €12.00 and gets the gummibears.

<u>Please ask questions NOW if anything remains unclear.</u>

You will have a chance to practice this task for 5 rounds that will not count towards determining your final earnings.

Please let the experimenter know that you finished reading.

Comprehension questions: (Choose all that apply)

1. *Suppose that in the round that is selected for payment you entered a* ***bid of €4*** *for a bag of chips. The randomly selected actual price turns out to be* ***€3****. What happens? Choose all that apply.*

    a) *I buy the chips for €4*
    b) *I buy the chips for €3*
    c) *My earnings in cash are €10.50*
    d) *My earnings in cash are €14.50*
    e) *My earnings in cash are €11.50*
    f) *I don't buy the chips*

2. *Suppose that in the selected round you had* ***bid €1*** *for a pack of crackers****.*** *The randomly selected actual* ***price*** *turns out to be* ***€2.75****. What happens? Choose all that apply.*

    a) *I buy the crackers for €2.75*
    b) *I buy the crackers for €1*
    c) *My earnings in cash are €14.50*
    d) *My earnings in cash are €11.25*
    e) *My earnings in cash are €13.50*
    f) *I don't buy the crackers*

3. *Suppose that in the round that is selected for payment,* ***you bid €3.20*** *for a bag of peanuts.* ***The actual price turns out to be €3.20****. What happens? Choose all that apply.*

    a. *I buy the peanuts for €3.20*
    b. *I buy the peanuts for €2.50*
    c. *My earnings in cash are €11.30*
    d. *My earnings in cash are €12.00*
    e. *My earnings in cash are €14.50*
    f. *I don't buy the peanuts*
