# Supplementary material for: The Fox and the Grapes—How Physical Constraints Affect Value Based Decision Making
Source: PLoS One. 2015 Jun 10;10(6):e0127619. doi: 10.1371/journal.pone.0127619 (PMC4464737; doi:10.1371/journal.pone.0127619)
Supplement: S2 Fig — The other pages of the instructions were identical to the physical condition. (PDF) [file pone.0127619.s003.pdf]

Instructions

Task
In this experiment, you will have an opportunity to buy a snack food from our store using €14.50 that you receive from us. You will receive the €14.50 once you have read the instructions and answered the comprehension questions correctly.

At the end of the experiment, you will be asked to stay in an adjacent room for 30 minutes. During this time, the only food that you will be allowed to eat is whatever snack you bought from us during the experiment.

Please note: All of the snack food items that are available in this experiment are regular size, as available in the supermarket. They have been recently purchased for the purpose of this experiment.

Your task in this experiment is to decide and tell us the maximum amount that you would currently be willing to pay for each of these items.

Round structure
The experiment consists of many rounds, all of which have a similar structure. In each round:

1. On the screen you will see the name and a description of the food item that is on offer in this round.
2. Then you will enter the maximum amount that you are willing to pay for this item (in €). You will need to enter a number between €0 and €4. You can also enter decimal numbers like €3.47 using the dot as a decimal point.
3. You will answer a few questions about the item.

There is no strict time limit for giving an answer. Nevertheless, try to answer spontaneously, without thinking too much. After 22 of these rounds there will be a break, then there will be another 22 rounds. You will see each item only once.

During this task a wristband will be attached to each of your arms.
