# Supplementary figures and images for: The Fox and the Grapes—How Physical Constraints Affect Value Based Decision Making
Source: PLoS One. 2015 Jun 10;10(6):e0127619. doi: 10.1371/journal.pone.0127619 (PMC4464737; doi:10.1371/journal.pone.0127619)

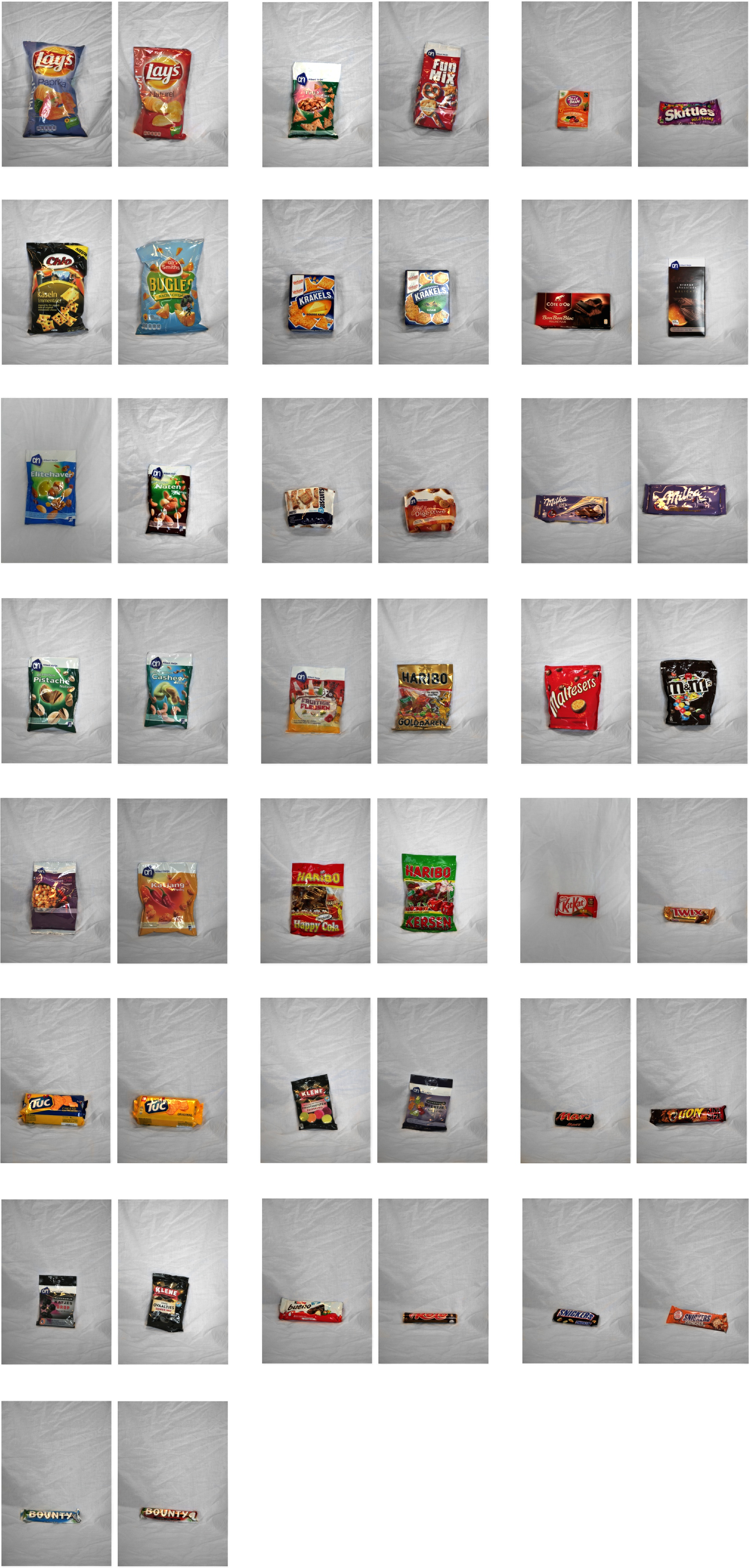

Supplement: S3 Fig — Pictures of the snack food items used in the physical condition. Snack foods near to each other comprise pairs of similar items (e.g. two chocolate bars or two licorice snacks). Elements of a pair were never presented in the same condition within one participant. One was presented in the weight, the other one in the no weight condition. This way, it was assured that there were the same kind of snack foods present in both conditions. Which item was presented in which condition was randomized across participants. (TIFF) [file pone.0127619.s004.tiff]

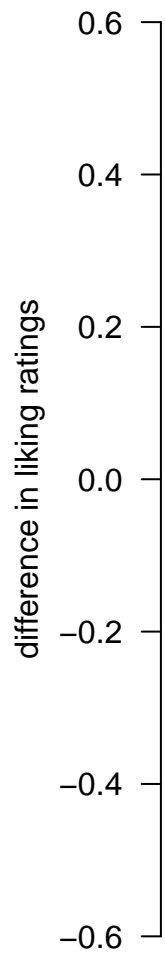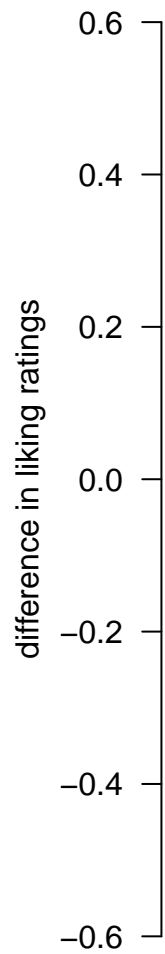

Supplement: S4 Fig — Each bar shows the average difference in liking of one participant across weight conditions. Negative values indicate that participants gave a lower average liking rating for items in the weight condition. (PDF) [file pone.0127619.s005.pdf]

difference in wanting ratings

a

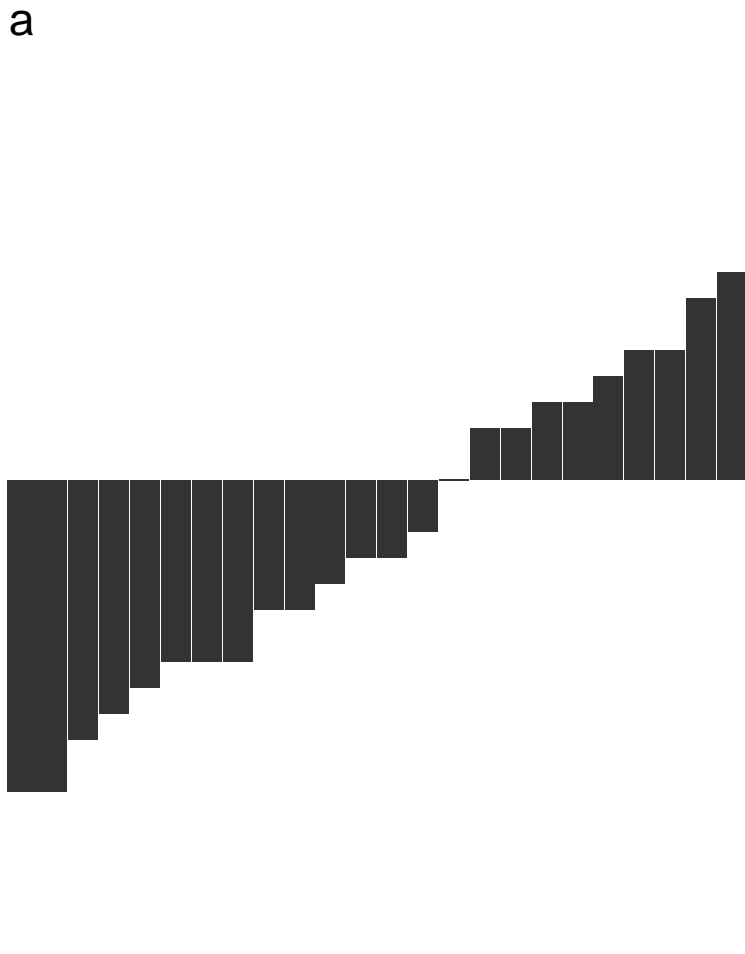

difference in wanting ratings

b

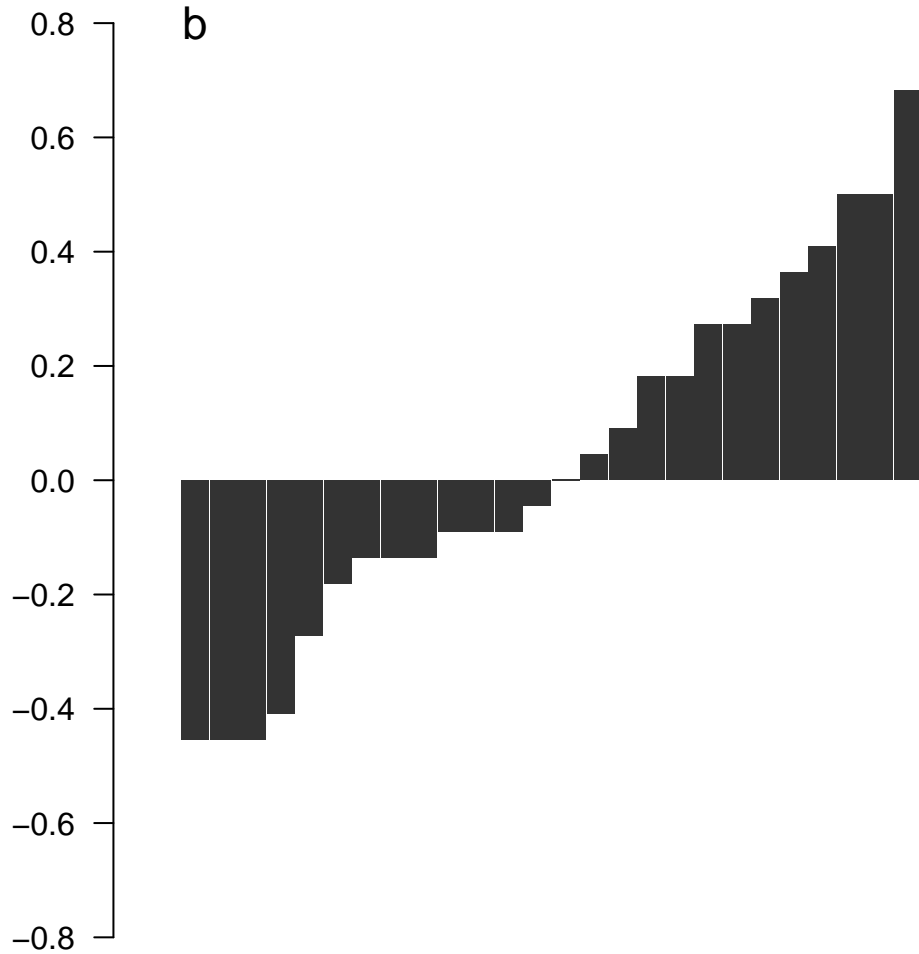

Supplement: S5 Fig — Each bar shows the average difference in wanting of one participant across weight conditions. Negative values indicate that participants gave a lower average wanting rating for items in the weight condition. (PDF) [file pone.0127619.s006.pdf]

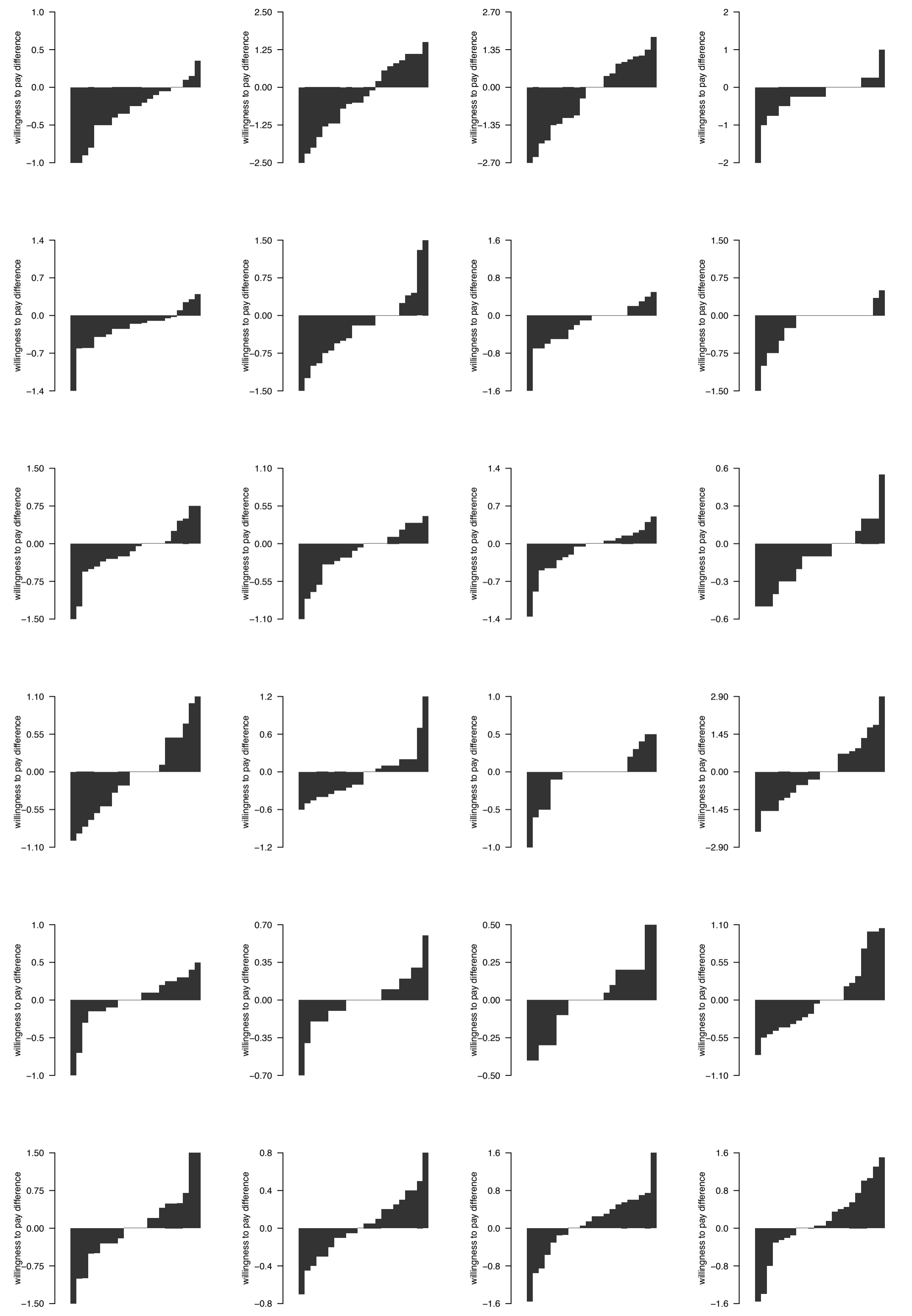

Supplement: S6 Fig — Each barplot shows the willingness to pay differences of one subject in the physical condition. Each bar represents the difference in willingness to pay for one item pair. As part of our item randomization strategy, we formed pairs of similar items. Within one subject, items from the same pair were never presented in the same condition. Thus, it is possible to compute for each subject the difference in WTP across the two items of each pair. It should be noted that, although items of the same pair were of the same type of snack food, they were still different, and any strong individual preferences (e.g. for or against cheese flavor on crisps, for or against orange flavor on chocolate, for or against raisins) still play a role. (PDF) [file pone.0127619.s007.pdf]

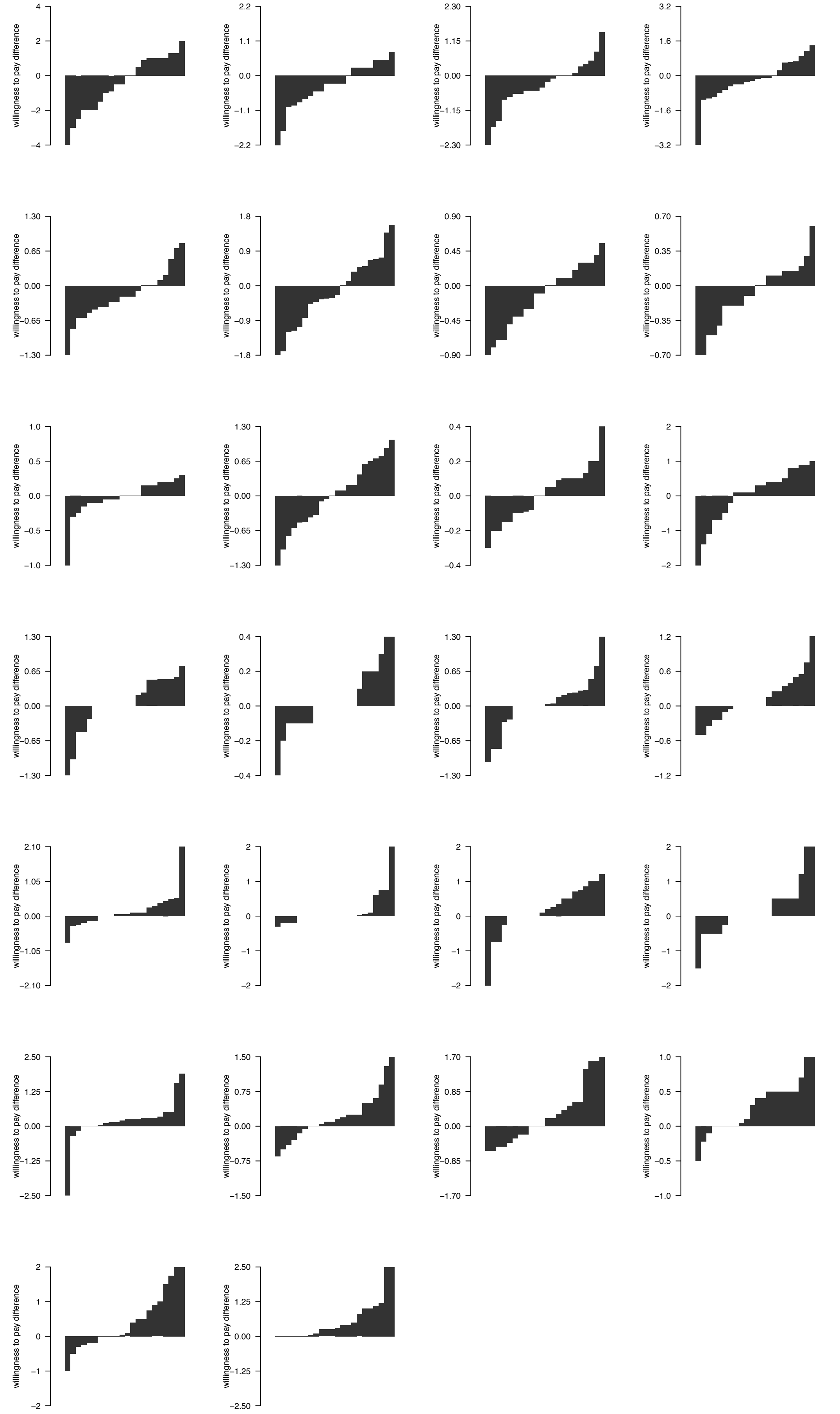

Supplement: S7 Fig — Each barplot shows the willingness to pay differences of one subject in the computer condition. Each bar represents the difference in willingness to pay for one item pair. As part of our item randomization strategy, we formed pairs of similar items. Within one subject, items from the same pair were never presented in the same condition. Thus, it is possible to compute for each subject the difference in WTP across the two items of each pair. It should be noted that, although items of the same pair were of the same type of snack food, they were still different, and any strong individual preferences (e.g. for or against cheese flavor on crisps, for or against orange flavor on chocolate, for or against raisins) still play a role. (PDF) [file pone.0127619.s008.pdf]
