# Supplementary material for: The Fox and the Grapes—How Physical Constraints Affect Value Based Decision Making
Source: PLoS One. 2015 Jun 10;10(6):e0127619. doi: 10.1371/journal.pone.0127619 (PMC4464737; doi:10.1371/journal.pone.0127619)
Supplement: S1 File — (PDF) [file pone.0127619.s009.pdf]

Supporting Information on Hypothesis Tests / Regression Models

In this section we elaborate on our choice of analysis strategy, and on the underlying assumptions. In the experimental psychology literature, it is common to aggregate data on the subject level. For comparison, we therefore present the results of t-tests as well as of an ANOVA analysis, both of which rely on subject-level averages. Below we detail the assumptions underlying the regression model that we report in the paper.

t-Tests

The simplest way to test our hypothesis that wristbands with weight decrease the valuation for snack food items in the physical but not in the computer condition is by computing the average willingness to pay, liking and wanting for each subject in each of the weight conditions, and compare these with a paired t-test separately for the physical and the computer condition. This does not, however, test directly whether the effect of the weights is different between the computer condition and physical condition.

In the physical condition participants showed a significant decrease in willingness to pay (paired samples t-test, t(23) = 3.74, p < .01, two-sided), while in the computer condition, average willingness to pay under heavy weights was not significantly different from average willingness to pay under no weight (paired samples t-test, t(25) = -0.81, p = .43, two-sided).

The same analysis can be applied to liking and wanting ratings. Liking ratings were marginally lower in the physical condition when wearing heavy wristbands (paired samples t-test, t(23) = 1.98, p = .06, two-sided). This was not the case in the computer condition (paired samples t-test, t(25) = -0.47, p = .64, two-sided).

A similar result was obtained for wanting ratings (physical condition: paired samples t-test, t(23) = 1.85, p = .08, two-sided; computer condition: paired samples t-test, t(25) = -0.53, p = .60, two-sided).

Analysis of Variance

By comparing the data in the aforementioned way we are not able to test the interaction of reachability × physical effort directly and we cannot control for the order of the weight conditions. This is possible when using Analysis of Variance (ANOVA). As for the t-tests, data was aggregated on the subject level. Thus for each subject we calculated the average willingness to pay, liking and wanting for each weight condition and each participant.

The ANOVA model contained one within subject factor (anticipated effort: no weight vs. weight condition), two between subject factors (reachability condition: physical vs. computer condition and order: starting the experiment with no weight or starting the experiment with weight) as well as the covariate familiarity (aggregated on subject-level) and all possible interactions of the main factors. As can be seen in Table S4, the analysis for willingness to pay revealed a significant interaction of reachability (physical vs. computer) × physical effort (no weight vs. weight). For the wanting and liking ratings as dependent variable, as already indicated by the t-tests, the interaction of reachability × physical effort did not reach the significance threshold of p < .05 in the ANOVA models (Table S5 and Table S6).

Table S4

Repeated measures analysis of variance.
Dependent variable: willingness to pay.

	df	SS	MS	F	p
**within subjects**					
anticipated effort	1	0.01	0.01	0.01	0.93
familiarity	1	1.59	1.59	2.81	0.10
order	1	0.98	0.98	1.74	0.19
anticipated effort × order	1	0.43	0.43	0.76	0.39
error	45	25.40	0.56		
**between subjects**					
reachability	1	0.02	0.01	1.10	0.30
familiarity	1	0.09	0.09	5.56	0.02
anticipated effort × reachability	1	0.08	0.08	4.75	0.03
reachability × order	1	0.02	0.02	0.88	0.35
anticipated effort × reachability × order	1	0.01	0.01	0.39	0.54
error	45	0.76	0.02		

Note. 50 subjects. Data aggregated over items, two data points for each subject. Within-subject factor: Anticipated effort (no weights vs. weights). Between-subject factor: reachability (physical vs. computer screen). All p values are two-sided.

Table S5

Repeated measures analysis of variance.
Dependent variable: wanting ratings.

	df	SS	MS	F	p
**within subjects**					
anticipated effort	1	0.04	0.04	0.16	0.69
familiarity	1	1.46	1.46	6.54	0.01
order	1	1.87	1.87	8.37	< 0.01
anticipated effort × order	1	0.34	0.34	1.53	0.22
error	45	10.05	0.22		
**between subjects**					
reachability	1	0.03	0.03	0.69	0.41
familiarity	1	0.22	0.22	5.72	0.02
anticipated effort × reachability	1	0.07	0.07	1.81	0.19
reachability × order	1	0.15	0.15	3.78	0.06
anticipated effort × reachability × order	1	0.08	0.08	1.96	0.17
error	45	1.75	0.04		

Note. 50 subjects. Data aggregated over items, two data points for each subject. Within-subject factor: Anticipated effort (no weights vs. weights). Between-subject factor: reachability (physical vs. computer screen). All p values are two-sided.

Table S6
Repeated measures analysis of variance.
Dependent variable: liking ratings.

	df	SS	MS	F	p
**within subjects**					
anticipated effort	1	0.05	0.05	0.22	0.64
familiarity	1	2.73	2.73	11.13	< 0.01
order	1	0.43	0.43	1.74	0.19
anticipated effort × order	1	0.72	0.72	2.92	0.09
error	45	11.03	0.25		
**between subjects**					
reachability	1	0.03	0.03	1.44	0.24
familiarity	1	0.16	0.16	6.78	0.01
anticipated effort × reachability	1	0.06	0.06	2.32	0.13
reachability × order	1	0.12	0.12	4.90	0.03
anticipated effort × reachability × order	1	0.01	0.01	0.59	0.45
error	45	1.08	0.02		

Note. 50 subjects. Data aggregated over items, two data points for each subject. Within-subject factor: Anticipated effort (no weights vs. weights). Between-subject factor: reachability (physical vs. computer screen). All p values are two-sided.

Regression Analysis

By aggregating the data on the subject-level, we unnecessarily reduce the amount of information (e.g. on within-subject variability), and we cannot control for familiarity on an item by item basis.

For this reason we fitted random intercept regression models to the data as described in the manuscript. In using this model, we make the following assumptions: We assume that observations belonging to different subjects are independent, that is we assume that the residual error term is uncorrelated across individuals. In contrast to a regression model with normal standard errors, we do not assume that observations have equal variance. For example, some subjects might vary their bid substantially from item to item, and others very little. We do also not assume that the residual error term is independent within individuals. That is, even after accounting for the fact that some subjects may bid higher than others with a random intercept, we may still expect some form of dependency across the observations belonging to the same subject. This could for example arise if subjects made a mistake on one trial, and reacted to that mistake by changing bidding behavior, or if subjects made their bids for items dependent on the previously encountered items. We account for these possible dependencies within subjects, as well as for possible heteroscedasticity, by using cluster-robust standard errors (Rogers, 1993). An additional requirement when using cluster-robust standard errors is that the number of clusters (in our case subjects) needs to be sufficiently large. With 50 clusters of equal size our dataset is large enough for accurate inference with this method (Kezdi, 2004; Miller and Cameron, 2013). The model is estimated using generalized least squares.

Model Diagnostics

Figure S8 to Figure S10 show diagnostic statistics of the fitted models. Figure Figure S8a reveals that there is indeed some heteroscedasticity in the willingness to pay data, with larger residuals for higher predicted values. As outlined above, this is accounted for by using cluster-robust standard errors. Figure Figure S8b suggests that residuals are identically distributed across the experimental conditions.

For liking and wanting, some effect of limiting the scale from 1-4 is evident in the residuals in Figures S9a and S10a, but no striking differences appear across the experimental conditions (see Figures S9b and S10b).

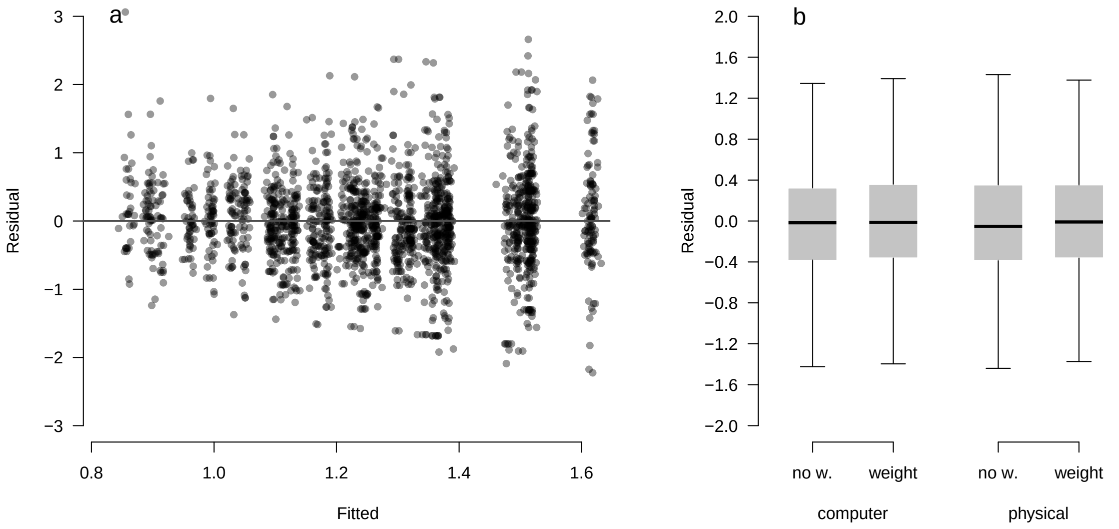

Figure S8. Residual plots of the willigness to pay random intercept regression. (a) Residuals vs. fitted values (slightly jittered) and (b) residual distribution across conditions.

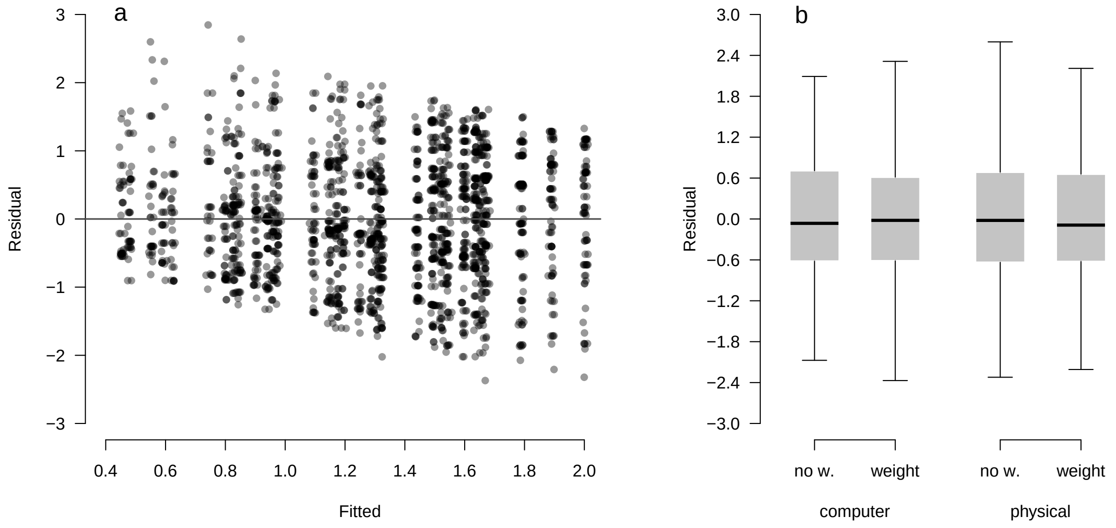

Figure S9. Residual plots of the wanting ratings random intercept regression. (a) Residuals vs. fitted values (slightly jittered) and (b) residual distribution across conditions.

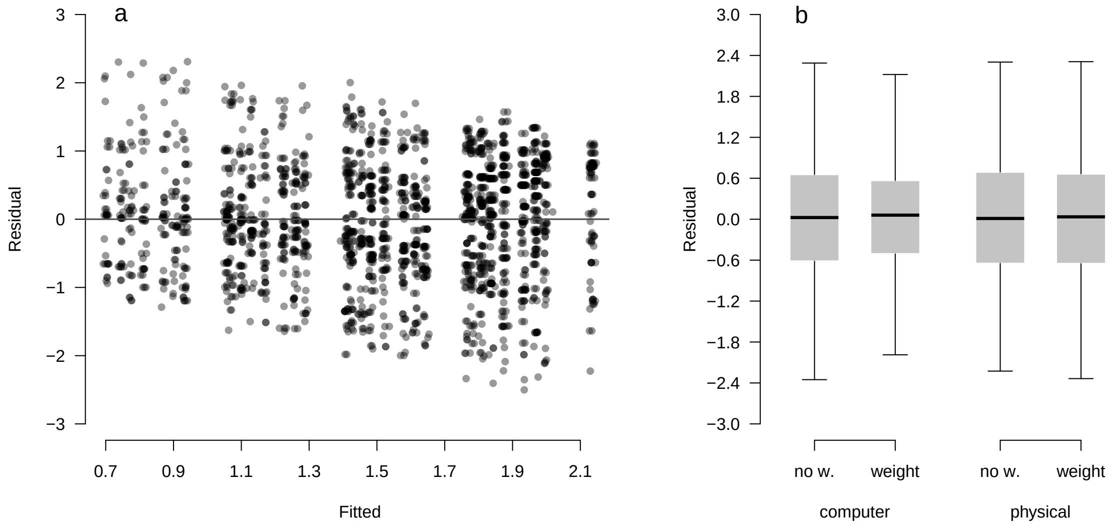

Figure S10. Residual plots of the liking ratings random intercept regression. (a) Residuals vs. fitted values (slightly jittered) and (b) residual distribution across conditions.

References

Kezdi, G. (2004). Robust standard error estimation in fixed-effects panel models. *Hungarian Statistical Review*, 9:96–116.

Miller, D. L. and Cameron, C. A. (2013). A practitioner's guide to cluster-robust inference. Working paper, University of California, Davis.

Rogers, W. H. (1993). Regression standard errors in clustered samples. *Stata Technical Bulletin*, 13:19–23.
