# Supplementary material for: The Fox and the Grapes—How Physical Constraints Affect Value Based Decision Making
Source: PLoS One. 2015 Jun 10;10(6):e0127619. doi: 10.1371/journal.pone.0127619 (PMC4464737; doi:10.1371/journal.pone.0127619)
Supplement: S1 Table — Names and descriptions (in parentheses) were presented together on the computer screen. Snack foods in the same row comprise pairs. (PDF) [file pone.0127619.s010.pdf]

Table S1
Stimuli used in the computer condition. For each item, names and descriptions (in parentheses) were presented together on the computer screen. Snack foods in the same row comprise pairs.

**Name**	**Name**
Digestive Mini's Milk Chocolate*<br>(Cookies)	Choc Chip Mini's*<br>(Cookies)
Salty Liquorice<br>(Liquorice)	Sweet Liquorice<br>(Liquorice)
Soft Fruitbears<br>(Fruitgum)	Fruity Winegums<br>(Fruitgum)
Haribo Happy Cola<br>(Fruitgum)	Haribo Happy Cherries<br>(Fruitgum)
Haribo Gummibears<br>(Fruitgum)	Fruity Bottles<br>(Fruitgum)
Jelly Beans<br>(Fruit Candy)	Skittles<br>(Fruit Candy)
Bounty<br>(Chocolate Bar)	Bounty Dark<br>(Chocolate Bar)
Cote d'or BonBonBloc Praline<br>(Chocolate)	Bitter Chocolate with Orange<br>(Chocolate)
Kinder Bueno<br>(Chocolate Bar)	Rolo<br>(Chocolates with Caramel)
KitKat<br>(Chocolate Bar)	KitKat Chunky*<br>(Chocolate Bar)
Choco M&Ms<br>(Chocolate Candy)	Maltesers<br>(Chocolate Candy)

Note. *These items were not identical but close substitutes to items in the physical condition, all other items were identical across the two conditions.

Table S1 (continued)

Stimuli used in the computer condition. For each item, names and descriptions (in parentheses) were presented together on the computer screen. Snack foods in the same row comprise pairs.

**Name**	**Name**
Mars<br>(Chocolate Bar)	Snickers<br>(Chocolate Bar)
Milka Milk<br>(Chocolate)	Milka Cream<br>(Chocolate)
Twix<br>(Chocolate Bar)	Lion<br>(Chocolate Bar)
Bugles Cheese<br>(Nacho Chips)	Chio Cheese<br>(Potato Chips)
Lay's Paprika<br>(Potato Chips)	Lay's Natural<br>(Potato Chips)
Fun Mix<br>(Salty Crackers)	Trio Zoutjes<br>(Salty Crackers)
Kaas Zoutjes*<br>(Cheese Crackers)	Snack Zoutjes*<br>(Salty Crackers)
Tuc Cheese<br>(Crackers)	Tuc Naturel<br>(Crackers)
Thai Sweet Chili Nuts<br>(Spicy Peanuts)	Katjang Pedis<br>(Spicy Peanuts)
Cashews<br>(Nuts)	Pistachios<br>(Nuts)
Nut and Raisins Mix<br>(Nuts)	Nut Mix<br>(Nuts)

Note. *These items were not identical but close substitutes to items in the physical condition, all other items were identical across the two conditions.
