# Supplementary material for: The Fox and the Grapes—How Physical Constraints Affect Value Based Decision Making
Source: PLoS One. 2015 Jun 10;10(6):e0127619. doi: 10.1371/journal.pone.0127619 (PMC4464737; doi:10.1371/journal.pone.0127619)
Supplement: S2 Table — Dependent variable: wanting ratings. (PDF) [file pone.0127619.s011.pdf]

Table S2

Random intercept regression model with control variables.
Dependent variable: wanting ratings.

	**Coef.**	**95% CI**	**p**
constant (computer condition, no weight)	0.45	[0.30, 0.61]	< 0.01
physical condition	0.10	[-0.11, 0.32]	0.35
weight	0.14	[-0.00, 0.28]	0.06
physical × weight	-0.21	[-0.41, -0.02]	0.03
familiarity	0.35	[0.28 ,0.41]	< 0.01
order of weight condition	0.29	[0.02, 0.57]	0.04
order × weight	-0.26	[-0.47, -0.05]	0.01
order × physical	0.11	[-0.30, 0.53]	0.59
order × physical × weight	0.22	[-0.08, 0.52]	0.15
σ_u (SD between subjects)	0.31		
σ_e (SD within subjects)	0.88		

Note. 2200 trials, nested within 50 subjects. Standard errors are corrected for potential heteroscedasticity and autocorrelations at the subject level. All p values are two-sided. Order indicates whether participant started with the weight (Order = 1) or no weight condition (Order = 0).
