# Supplementary material for: The Fox and the Grapes—How Physical Constraints Affect Value Based Decision Making
Source: PLoS One. 2015 Jun 10;10(6):e0127619. doi: 10.1371/journal.pone.0127619 (PMC4464737; doi:10.1371/journal.pone.0127619)
Supplement: S3 Table — Dependent variable: liking ratings. (PDF) [file pone.0127619.s012.pdf]

Table S3

Random intercept regression model with control variables.
Dependent variable: liking ratings.

	**Coef.**	**95% CI**	**p**
constant (computer condition, no weight)	0.81	[0.65, 0.98]	< 0.01
physical condition	-0.07	[-0.32, 0.19]	0.60
weight	0.09	[-0.02, 0.20]	0.11
physical $\times$ weight	-0.13	[-0.29, 0.02]	0.08
familiarity	0.35	[0.30, 0.41]	< 0.01
order of weight condition	0.06	[-0.23, 0.34]	0.70
order $\times$ weight	-0.19	[-0.33, -0.04]	0.01
order $\times$ physical	0.27	[-0.13, 0.67]	0.19
order $\times$ physical $\times$ weight	0.09	[-0.14, 0.33]	0.44
σ_u (SD between subjects)	0.33		
σ_e (SD within subjects)	0.85		

Note. 2200 trials, nested within 50 subjects. Standard errors are corrected for potential heteroscedasticity and autocorrelations at the subject level. All p values are two-sided. Order indicates whether participant started with the weight (Order = 1) or no weight condition (Order = 0).
